# Gender Differences in the Association of Hazardous Alcohol Use with Hypertension in an Urban Cohort of People Living with HIV in South Florida

**DOI:** 10.1371/journal.pone.0113122

**Published:** 2014-12-09

**Authors:** María José Míguez-Burbano, Clery Quiros, John E. Lewis, Luis Espinoza, Robert Cook, Allison B. Trainor, Erika Richardson, Deshratn Asthana

**Affiliations:** 1 School of Integrated Science and Humanity (SISH), Florida International University, Miami, Florida, United States of America; 2 Department of Psychiatry and Behavioral Sciences, Miller School of Medicine, University of Miami, Miami, Florida, United States of America; 3 Division of Infectious Diseases, Miller School of Medicine, University of Miami, Miami, Florida, United States of America; 4 Department of Epidemiology, College of Public Health and Health Professions & College of Medicine, University of Florida, Gainesville, Florida, United States of America; 5 Department of Psychiatry and Behavioral Sciences, Department of Pathology, Miller School of Medicine, University of Miami, Miami, Florida, United States of America; Stellenbosch University, South Africa

## Abstract

**Objective:**

Industrialized countries are currently experiencing an epidemic of high blood pressure (HBP) extending to people living with HIV (PLWH). Given the prevalence of hazardous alcohol use (HAU), this study examines the relationship between alcohol consumption and hypertension in PLWH. Including a gender analysis is critical, given the high rates of HAU and HIV among females.

**Method:**

We followed PLWH including both HAU and non-HAU (200 each). Participants were assessed twice for body weight, blood pressure, alcohol consumption, and other BP-associated lifestyle factors. High blood pressure (defined as systolic/diastolic blood pressure above 140/90 mmHg and/or treatment of HBP) was the primary outcome.

**Results:**

Overall prevalence of hypertension was 38% and higher among HAU compared to non-HAU (42% vs. 34%, *p* = 0.02). Less than half with HBP (42%) were receiving treatment for hypertension. Overall, males had a 50% higher risk of HBP than women (odds ratio: 1.5, 95% CI: 1–2.6, *p* = 0.05). However among HAU, females were twice as likely to suffer HBP as their male counterparts (95% CI: 1–3.9, *p* = 0.02). Those HAU who preferred liquor, versus wine, had higher adjusted mean BP (132.6±18 vs. 122.3±14 mm Hg, *p* = 0.05). Additional analyses indicated that consumption of >1 standard drink of liquor or beer/day was associated with HBP. Risk of hypertension was noted in those with daily consumption of >3 glasses of wine. For those reporting <1 drink per day, the odds ratio of having HBP was 0.97 (CI: 0.6–0.99, p = 0.05). Factors associated with hypertension in the multivariate model included increased age, gender, BMI, HAU particularly of liquor, and smoking.

**Conclusions:**

Excessive hypertension burden in this population and its association with HAU and sub-optimal care indicate the need for preventive and educational intervention in PLWH. Analyses highlight the necessity of gender and type-of-beverage specific approaches.

## Introduction

After the increased longevity achieved with the success of antiretroviral therapy (ART), HIV experts are now concerned with the increased risks of cardiovascular diseases (CVD) observed among people living with HIV (PLWH). For PLWH, rates of CVD are approximately two-fold higher than for age-matched people without HIV infection. [1]–[2] Nonetheless, studies evaluating weather hypertension is more prominent among PLWH have reached controverting conclusions [3]–[7]. The contradictory findings in the burgeoning literature are likely related not only to different study designs or populations, but also the accrual of risk factors.

Several epidemiological studies among the general population have documented a close relationship between alcohol consumption and systemic hypertension [9]. Indeed, a linear dose-response relationship has been identified [10]–[12]. In other studies, the relationship has been non-linear, especially in women. Notably, some authors have proposed that moderate consumption of alcohol may be cardioprotective by reducing blood pressure [13]. Nonetheless, some methodological limitations exist. Few studies were longitudinal, and data regarding the plausible effects of the type of alcoholic beverage have been insufficiently assessed [14]. Many of these studies have failed to analyze body composition, although it is known that hypertension commonly clusters with obesity [8]. While the association between HAU and HBP has been investigated exhaustively in populations across the globe, few studies have been conducted in PLWH.

Including women in this type of study is critical because HBP is a significant risk factor for CVD, which is the leading cause of death in women. Every minute in the United States, a woman dies from a CVD. Notably, while in younger adults (<45 years of age), HBP rates are higher among males than females, and with aging the proportion of women with hypertension starts rising and surpasses those of the male counterparts. [15] Given that the risk of CVD doubles for every 20 mmHg increase in systolic BP, or 10 mmHg increase in diastolic BP, hypertension could be a serious contributor to gender differences in morbidity and mortality. Therefore, analyses of gender differences regarding the prevalence, treatment, and control rates of hypertension are of public health significance.

This study aims to address these important issues by examining the relationships between alcohol consumption and HBP among men and women living with HIV. The PADS (Platelets Mediating Alcohol and HIV Damage Study) cohort provides a unique opportunity, not only because detailed information on alcohol use was collected, but also because of the large participation of women and minority individuals. In addition, PADS was heterogeneous regarding beverage preference and therefore an ideal cohort to obtain the most informative data regarding high-risk groups and correlates of risks. This information is necessary to develop effective prevention and treatment programs.

## Methods

### Sampling

The PADS is a large, single-site multi-ethnic cohort consisting of 400 PLWH, who are at least 18 years old and under regular care at Miami's primary open-access public health system. Recruitment and assessments took place in the period between June 2010 and June 2012. Our choice of PLWH in an open-access public health system with standard treatment protocols was purposefully designed to minimize social, medical, and treatment inequalities.

Males and females with confirmed HIV infection were eligible. Non-ambulatory patients, and those presenting with major medical co-morbidities such as major neuropsychological (i.e., active central nervous system opportunistic infection, tumors, developmental disorders), immune-based (i.e., malignancies, autoimmune diseases), and chronic diseases (i.e. renal failure) were not included. In addition, subjects who had cirrhosis, active viral hepatitis, or liver enzymes two standard deviations above normal values were not eligible. To reduce the confounding effects of illicit drug use, the DSM-IV-TR questionnaire was applied, and those individuals who were dependent on drugs or injecting illicit psychoactive substances were excluded.

### Participants' Assessment Protocol

PADS was approved by the Institutional Review Boards at Florida International University and University of Miami. The study was conducted according to the principles expressed in the Declaration of Helsinki. Those participants who provided written informed consent were consecutively enrolled and followed over a period of six months.

### Measures

#### Outcome Variable –Hypertension

Blood pressure was measured by a health care provider at the research center using a calibrated automated machine in an upright position. After the participant had rested at least five minutes, systolic and diastolic measurements were registered at the first and fifth Korotkoff sounds. Following national guidelines, two readings were performed at each visit using Sure sign VS2 (Phillips), and the average was recorded. Hypertension was defined using the World Health Organization (WHO)/International Society of Hypertension (ISH) guidelines: diastolic blood pressure (DBP) values of ≥90 mm Hg or systolic blood pressure (SBP) values of ≥140 mmHg at the two clinic visits (i.e., baseline and 6 months). Following the Current Joint National Committee 7 guidelines, HBP was further subdivided into: Stage 1 (SBP 140-159 or DBP 90–99 mm Hg) and Stage 2 (SBP >160 or DBP >100 mm Hg) [18]. Subjects being prescribed an antihypertensive medication, irrespective of blood pressure, were also defined as hypertensive. Data on prescribed medications and history of HBP were collected by using standardized questionnaires and confirmed with medical records when available.

#### Alcohol Use Profiling

Since the main focus of this cohort study was to assess the potential effects of alcohol, participants were dichotomized as hazardous and non-hazardous alcohol users. To classify participants, alcohol intakes were collected using the Alcohol Use Disorders Identification Test (AUDIT) and the Alcohol Dependence Scale (ADS) [16]–[17]. To isolate the impact of different types of alcohol consumption on the risk of HBP, participants were queried regarding their predominant type of alcoholic beverage (i.e., ≥75% of all times: beer, wine, or liquor). This information is highly relevant in light of prior publications indicating differential health effects by type of alcoholic beverage [19]–[20]. A standard drink was defined as 12 fluid ounces of beer, 5 fluid ounces of wine, or 1.5 fluid ounces of distilled spirits (equivalent to 0.5 ounces or 14 grams of alcohol). Alcohol consumption scores were computed by averaging cross products of quantity and frequency of beer/wine and hard liquor reported on the ADS responses.

Subsequently, based on the National Institute of Alcohol Abuse and Alcoholism guidelines criteria and using gender specific criteria, participants were classified as HAU, while those who reported fewer drinks were categorized as non-HAU. Specifically, men who reported >2 drinks per day (14 drinks/week) and women reporting more than one drink per day (or binge drinking >3 drinks in one day) were classified as HAU.

### Potential Confounders

Other measures included sociodemographics (i.e., gender, age, race/ethnicity, income, and education), smoking (the Fagerström Test for Nicotine Dependence), and total dietary intake (24 hour dietary intake). Basic body composition indices including weight and height to calculate body mass index (BMI; weight [lbs]/height [inches]^2^ ×703) were obtained.

Since it is not clear whether HIV or a specific ART is an independent risk factor for hypertension, we quantified CD4 counts and HIV viral load (Amplicor HIV monitor test, Roche Diagnostic System). We also documented ART and adherence using both self-report and medical chart abstraction (ACTG questionnaire).

### Statistical Analyses

The data were analyzed using SPSS 20 (IBM, Inc., Chicago, IL), and *p* values <0.05 were considered to be statistically significant. Means were compared using Student's t-test and one-way analysis of variance (ANOVA) procedures. Univariate analyses were used to calculate odds ratios (OR) and 95% confidence intervals (CI). Logistic regression analyses were used to evaluate the effects of alcohol (continuous or as hazardous vs. non-hazardous), BMI (continuous and dichotomized as >30), age (continuous and dichotomized at age 40), and gender on BP.

The multivariate model that examined factors associated with HBP included all covariates that were significant in the univariate models plus other potential predictors (i.e., age, gender, race/ethnicity, CDC HIV disease status, BMI, alcohol use, and ART) selected on the basis of the HIV medical literature. More parsimonious models were explored by removal of covariates, one at a time, starting with the covariate with the largest *p value*, until the final full model was achieved.

## Results

The mean SBP of the group at baseline was 122.9±15.2 mm Hg and the mean DBP was 77±11. At the last visit, SBP was 123.5±16 mm Hg and DBP was 78.6±10 mm Hg. The prevalence of hypertension in our study cohort was high with 38% having HBP. Less than half of those with repetitive blood pressure readings above the recommended limits (140/90 mm Hg) at baseline (45%) and at the last visit (48%) were receiving treatment. However, an additional 20% were prescribed a medication and were not taking it.

### Bivariate analyses

As in the general population, hypertensive subjects were older. Significant differences by age groups were evident between persons > age 40 compared to age 40 and younger for both SBP (124.1±15.7 vs. 118.8±12.8 mm Hg, *p* = 0.004) and DBP (78.1±11.3 vs. 75.2±9.7 mm Hg, *p* = 0.04). As depicted in Table 1 analyses uncovered gender differences, including in mean SBP (men  = 123.9±14 vs. women  = 121.0±16.3 mm Hg, *p* = 0.07). Overall, males had a 50% higher risk of HBP than women (OR: 1.5, 95% CI: 1–2.6, *p* = 0.05). Hypertensive women were less likely to be receiving treatment for their HBP (OR = 0.76, 95% CI: 0.4–1.1, *p* = 0.05).

**Table 1:** Sociodemographic and Clinical Characteristics of HIV-infected Patients by Gender.

Variable	Male	Females = 110	P value
HIV Diagnosis Year (mean)^a^	16±7	18±7	0.09
Age (years)^a^	42.3±6.9	42.8±5.7	0.44
BMI kg/m^2b^	26.6±6.0	31.7±8.6	0.00
Albumin mg/dl^b^	4.2±0.5	4.1±0.4	0.01
CD4 Cell Counts^b^	386.3±257	508.7±315	0.00
Viral Load (log)^b^	2.8±1.3	2.7±1.4	0.30
Systolic Pressure mmHg	123.9±14.5	121.0±16.3	0.07
Diastolic Pressure mmHg	77.3±10.5	77.7±11.8	0.79
Total drinks per week	18.7±2	12.7±2	0.02

a Demographic characteristics were expressed as percentages by gender groups.

b Biological measures were presented as means and standard deviations.

Compared to eutrophic subjects, obese individuals had the highest mean SBP, both at baseline (127.4±14 vs. 121.2±16.2 mm Hg, *p* = 0.0001) and at the follow-up visit (129.1±13 vs. 120.3±19 mm Hg, *p* = 0.001). DBP also differed between obese and non-obese individuals (81.4±9 vs. 76.1±10, *p* = 0.001). They were also more likely to be HAU (82%) than others. Next, we assessed the prevalence of hypertension across categories of BMI and found that obese individuals were 4 times more likely to have stage 2 hypertension (95% CI: 1–19.9, *p* = 0.04). Overweight individuals also had higher odds of HBP, when compared to eutrophic individuals (OR: 2.4, 95% CI: 1.3–4.3, *p* = 0.002).

### Blood Pressure and HIV-related Factors

Hypertensive and normotensive individuals were comparable in terms of numbers of years living with HIV infection. Given prior postulates that ART increases the risk for the development of hypertension, we explored this relationship [22]–[23]. However, the prevalence of HBP was similar between those receiving or not receiving ART (33% vs. 27%). Mean SBP was also similar between those receiving Truvada (121±15 vs. 124±16 mm Hg, *p* = 0.06), Norvir (122±16 vs. 123±14 mm Hg, *p* = 0.7), and Atripla (123±15 vs. 122±15 mm Hg, *p* = 0.7), versus those who did not. Neither was the number of years receiving ART (8 vs. 7 years, *p* = 0.2), so no further analyses were performed. We did not observe a significant relationship between duration of HIV infection and HBP [see Table 2].

**Table 2:** Baseline Sociodemographic Information of HIV-infected Patients by BP Groups.

Variable	Normotensive n = 260	Stage 1 Hypertension n = 110	Stage 2 Hypertension n = 30	P value
HIV Diagnosis Year (mean)^a^	17±1.4	17±1.8	18±1.3	0.9
Age (years)^a^	41.7±6.9	43.7±5.7	46.2±4.0	0.009
Men^a^	61%	69%	59%	0.3
Women^a^	39%	31%	41%	0.3
African American^a^	69%	70%	88%	0.5
Hispanic^a^	24%	24%	12%	0.5
White^a^	7%	6%	0%	0.5
Annual Income:^a^				
Less than $10,000	88%	87%	76%	0.7
$11,000–$49,000	9%	7%	17%	0.7
>$50,000	3%	6%	17%	0.7
BMI kg/m^2b^	27.6±6.7	30.1±8.3	31.6±9.8	0.09
Smoking (cigarettes/day)^b^	3.1±1.5	8.1±1.3	8.8±0.7	0.03
CD4 Cell Counts^b^	428±283	473.0±306	394±242	0.1
Viral Load (log)^b^	2.8±1.3	2.6±1.22	2.3±0.86	0.4
Systolic Pressure mmHg	120.5±14	143.5±4.8	168.9±11.9	0.000
Diastolic Pressure mmHg	75.6±13	90.1±4.6	96.3±22.6	

a Demographic characteristics were expressed as percentages by blood pressure groups.

b Biological measures were presented as means and standard deviations.

Though in the past longer duration of HIV infection has been associated with HBP, we failed to observe any significant difference [see Table 2]. As depicted in Table 2, subjects with stage 2 HBP had the lowest viral loads, but not to a significant extent. Supplementary analyses indicated that those who had achieved undetectable viral loads had significantly higher SBP (124.7±15.5 vs. 120.2±14.7 mm Hg, *p* = 0.005). The diastolic blood pressure was also different (78.4±11.8 vs. 75.9±9.7 mm Hg, *p* = 0.03).

Although baseline CD4 count as a continuous variable showed no significant associations with hypertension, when the sample was dichotomized above and below 200 CD4 cells, differences emerged. Subjects with CD4 counts >200 cells/mm^3^ were more likely to have HBP, (OR: 1.7, 95% CI: 1–3.1, *p* = 0.03).

### Alcohol Use and Blood Pressure

Though among non-HAU over half (55%) never or rarely drank alcoholic beverages, 34% imbibed at least once a week, typically more than two drinks per occasion. HAU were actively drinking at the time of assessment and imbibed in excess of 7±5 drinks per day. They regularly consumed alcohol an average of 4 days a week, and the mean total intake was 31 drinks per week. Table 3 shows the descriptive characteristics of the total sample by alcohol status. As depicted in Table 1, HAU were more likely to be smokers. Smokers had a 60% higher risk of having HBP (OR: 1.6, 95% CI 1–2.5, *p* = 0.01). Notably, HAU that smoked almost doubled their risk (OR: 2.6, 95% CI 1.3–5.14, *p* = 0.002).

**Table 3:** Sociodemographic and Clinical Characteristics of HIV-infected Patients by Alcohol Groups.

Variable	HAU N = 198	Non-HAU N = 199	P value
Age (Years)^a^	43±6.4	41±6	0.7
Men^a^	67%	60%	0.1
Women^a^	33%	40%	0.1
African American^a^	70%	66%	0.4
Black Caribbean^a^	2%	4%	0.4
Hispanic^a^	22%	23%	0.4
White^a^	6%	7%	0.4
Annual Income:^a^			
Less than $10,000	88%	86%	0.7
$11,000–$20,000	8%	10%	0.7
$20,000–$49,000	2%	2%	0.7
>$50,000	2%	2%	0.7
Education (years of school)^a^	11.5±2	11.3±2.4	0.1
Albumin mg/dl^b^	4±0.4	4.1±0.5	0.9
Total Caloric Intake (Kcal/day)^b^	2379.6±1214	2042.1±968	0.1
BMI kg/m2^b^	29.3±7.9	27.6±7.0	0.03
CD4 Cell Count^b^	408±259	455±310	0.1
Viral Load (Log)^b^	2.7±1.3	2.6±1.3	0.4
Systolic Pressure mmHg	124.5±14.7	121.120±15.6	0.03
Diastolic Pressure mmHg	78.3±10.1	76.6±11.8	0.2

a Demographic characteristics were expressed as percentages by alcohol groups (except for Education).

b Biological measures were presented as means and standard deviations.

Overall prevalence of hypertension was 38% and higher among HAU compared to non-HAU (42% vs. 34%, *p* = 0.02). The SBP of HAU was significantly higher than that of non-HAU (124.5±14 vs. 121.2±15.6 mm Hg, *p* = 0.04), yet the DBP was only marginally higher (78.3±10.1 vs. 76.7±11.8 mm Hg, *p* = 0.09). HAU exhibited higher odds of HBP compared with non-HAU (OR: 1.4, 95% CI: 1.03–1.87, *p* = 0.01). Gender analyses revealed, that among HAU, women were twice as likely to have HBP, compared to their male counterparts (95% CI: 1–3.9, *p* = 0.02). In addition, HAU females exhibited much higher OR of having HBP than the non-HAU women (OR: 3, 95% CI: 1.3–5.8, *p* = 0.004).

The number of drinks per day was related to blood pressure. Subjects drinking more than 1 drink per day exhibited significantly higher systolic and diastolic blood pressures, compared with those drinking <1 drink per day, 3.6 mm Hg (systolic 123.95±14.8 vs. 120.3±15.9 mm Hg, *p* = 0.05 and diastolic 78.4±10.5 vs. 75.1±11.8 mm Hg, *p* = 0.05, respectively).

Since Marmot et al. discovered a significant relationship between drinking more than three drinks per day and HBP, a similar analysis was performed [21]. Similar to Marmot's study, drinkers of more than 3 drinks per day tended to have higher unadjusted risks of hypertension, compared with other drinking categories (OR: 1.2, 95% CI: 0.9–1.68, *p* = 0.07). The difference between the BP level of those consuming 1–3 drinks per day, compared with abstainers was 4.0 mm Hg (124.1±14.9 vs. 120.9±15.5, *p* = 0.05 mm Hg), yet DBP did not differ.

Type of beverage was also associated with blood pressure. Liquor use was reported by a third of the drinkers, beer by 45%, 22% reported preferences for wine and the remaining 10% indicated not preferences. For predominant beverage consumption, HAU liquor drinkers on average showed a higher SBP (130.2±13 vs. 122.6±15.5 mm Hg, *p* = 0.045), compared to non-liquor drinkers. On the other hand, no significant differences in SBP (123.8±14 vs. 119.0±13 mm Hg) or DBP (78.2±10 vs. 72.0±6.2 mm Hg) were found among HAU and non-HAU of other alcoholic beverages. The relationship between liquor and SBP remained significant after controlling for confounders (i.e., age, gender, race, diet, and physical activity). To gauge the extent to which the type of beverage was related to hypertension risk in a dose-response fashion, we sorted respondents according to the number of drinks consumed per occasion. If the subject consumed more than 3 cups of wine per day, then a significant deleterious effect of BP was observed (126±15 vs. 122.3±14 mm Hg, *p* = 0.05), but even in this case liquor drinkers exhibited the highest SBP values. No significant differences were evident among beer users.

Finally, a slightly higher proportion of HAU that were hypertensive were not taking any medication (58%) as compared to non-HAU in whom 50% were taking their medication.

### Multivariable Analyses

Since we aimed to assess the relationship among specific types of alcohol consumption, hypertension, and gender, we examined predictors of hypertension in 4 separate models (the first model in HAU: Table 4; the second in non-HAU: Table 5; in men: Table 6; and in women: Table 7). As illustrated in Table 3, in addition to receiving treatment, other factors significantly associated with hypertension among non-HAU were increasing age (*p* = 0.01), male gender (*p* = 0.02), higher BMI (*p* = 0.03), and liquor use (*p* = 0.01). In the second model for HAU, two distinct factors were significantly associated with hypertension: female gender and smoking. Notably in the gender models, alcohol, specifically liquor and smoking, remained as significant predictors only among females. Of interest, none of the HIV specific variables remained significant in any of the models.

**Table 4:** Multivariate Analyses among Non-hazardous Alcohol Users.

Model		Unstandardized Coefficients		Standardized Coefficients	t	P value
		B	Std. Error	Beta		
1	(Constant)	.732	.156		4.705	.000
	BMI	.062	.029	.113	2.136	.033
	Age 40	.135	.052	.128	2.571	.011
	Gender male	.111	.050	.114	2.204	.028
	HBP treatment^a^	.291	.060	.243	4.827	.000
	Liquor	−.022	.009	−.124	−2.488	.013

a Dependent Variable: Hypertension at the last visit (yes/no).

**Table 5:** Multivariate Analyses among Hazardous Alcohol Users.

Model			Unstandardized Coefficients		Standardized Coefficients	t	P value
		B	OR	Std. Error	Beta		
1	(Constant)	−.130	0.88	.190		−.682	.496
	BMI	.066	1.07	.026	.116	2.585	.010
	Age 40	.099	1.10	.047	.090	2.123	.034
	Gender male	.103	1.11	.045	.101	2.305	.022
	HBP treatment^a^	.668	1.95	.053	.533	12.488	.000
	Smoking	.108	1.13	.044	−.104	−2.449	.015
	Liquor	.146	1.16	.063	.099	2.326	.021

a Dependent Variable: Hypertension at the last visit (yes/no).

**Table 6:** Multivariate Analyses: Predictors of Blood Pressure among Men.

Model	Unstandardized Coefficients		Standardized Coefficients	t	P value
	B	Std. Error	Beta		
BMI	.433	.167	.176	2.593	.000
Age	.300	.137	.144	2.189	.010
HBP treatment^a^	−8.429	2.67	−.214	−3.154	.002
Number of cigarettes	−.058	.075	−.049	−.771	.442
Liquor	0.524	.338	.102	1.549	.123

a Dependent Variable: Systolic Blood Pressure as a Continuous Variable.

**Table 7:** Multivariate Analyses: Predictors of Blood Pressure among Women.

Model		Unstandardized Coefficients		Standardized Coefficients	t	P value
		B	Std. Error	Beta		
1	BMI	.433	.145	.238	2.975	.004
	Age	.555	.198	.223	2.804	.006
	HBP treatment^a^	−10.146	2.767	−.285	−3.667	.000
	Number of cigarettes	−.329	.131	−.194	−2.511	.013
	Liquor	1.087	.491	.171	2.216	.029
	Wine	−0.72	1.000	−.006	−.072	.943
	Beer	.255	.490	.042	.520	.604

a Dependent Variable: Systolic Blood Pressure at the last visit, as a Continuous Variable.

## Discussion

Our analyses revealed a prevalence of hypertension (38%) that is higher than previously reported for PLWH (8–34%) and age-matched rates in the general population (33%). Of concern, less than half of those with elevated BP readings in the study were receiving treatment. Noteworthy, another 20% were prescribed treatment but were not taking it, possibly because of the burden of pills and the usual asymptomatic nature of the disease. These rates are of great concern, given that hypertension places stress on the vasculature, kidneys, and heart, which could result in arrhythmia, heart failure, cardiomyopathy, valvular disease, kidney failure, and stroke. Therefore, understanding factors associated with hypertension, especially reversible behaviors such as smoking and drinking, has high public health relevance. In this regard, our data demonstrated that HAU is an important predictor of HBP. Furthermore, the data provide further directives, as results highlight the importance of limiting alcohol intake to one drink per day. Educating PLWH regarding the harms of HAU, particularly the risks of CVD, should be an important component of disease prevention. Overall, the model also suggests that the previously proposed role of ART on risk of HBP is either small or affects only a limited proportion of susceptible subjects. Our initial observation might be attributable to BMI, since HIV-related variables were no longer significant once we incorporated BMI and HAU into the model. These findings highlight the importance of focusing on primary prevention and therapy on subjects with alcohol, smoking, and obesity comorbidities. By doing so, the detection of other CVD risk factors can also be improved.

Our results indicated that outcomes appear to be gender specific, and therefore failure to conduct gender analyses can lead to incorrect conclusions. We found that males are at greater risk for developing hypertension than are age-matched women. Findings are in accord with prior studies among the general population, which have attributed disparities in hypertension rates to the protective effects of hormones [24]–[25].

Yet, our most important finding lies in discovering that HAU-women have higher rates of HBP, suggesting that they may be more susceptible to the vasopressor effects of alcohol. Differences could be attributed to the higher rates of obesity among hazardous drinking women, but not among men, in whom drinking was rather associated with low BMI. Obesity is known to exacerbate the risks of hypertension and could explain the observed gender differences. Finally, gender differences in treatment rates were also evident with women being less likely to be taking treatment for HBP. Though participants in our study were all involved in health care, it is possible that low income among women may impact their capability to obtain medication(s).

Equally important, our study provides significant evidence regarding the differential influences of type of alcoholic beverage on rates of systemic hypertension. After adjustment for key confounding factors, such as general health, diet, and social and economic variables, liquor use conferred greater BP risks than beer or wine in this sample. Of concern, SBP, which was particularly higher among liquor users, is a better predictor of risk for cardiovascular and renal disease than DBP [26]. Findings are consistent with prior publications [27]–[30]. Leighton and colleagues[27] attributed the vascular differences among liquor, beer, and wine to flavonoids and polyphenols, which enhance endothelial nitric oxide synthase expression and subsequent nitric oxide release from endothelial cells [27]–[28]. On the other hand, alcohol, which in this case equates to liquor (alcohol without antioxidants, resveratrol), has been associated with suppressed bioavailability of nitric oxide. In addition, antioxidants and other secondary components of wine and beer can also enhance flow-mediated vasodilatation [28]–[29]. Yet, it is important to remember that for those individuals consuming more than 3 drinks of wine or beer daily the beneficial effects disappear, probably because the balance between alcohol and antioxidants is lost. Yet, regardless of the mechanism(s), the public health and clinical message is clear: the use of alcohol is an important risk factor for hypertension, particularly if liquor is used.

Of concern is the confluence of HAU, obesity, and smoking, which at least doubled the risk for HBP in this population. These results highlight the need for global prevention approaches; otherwise the survival benefits of ART will be soon offset. Noteworthy, none of the HIV parameters (ART, CD4, or viral load) remained as significant predictors of hypertension. Findings are in line with large clinical trials, though others have argued that ART, low CD4, and viral load can influence the odds of hypertension. Because obesity could be associated with ART and better CD4 and viral responses, higher BMI will be the direct contributor to hypertension.

Our results need to be analyzed with some caveats. For example, our study is based on a single site cohort in Florida. Yet, results are consistent with national cohorts, as well as with scientific reports around the globe, which argue for the applicability of our findings. Second, while the collection of alcohol data was very detailed, alcohol intakes were obtained by self-reports only, which is considered a common drawback, given the risks of either under or over-estimation.

On the other hand, this study has several strengths. First, a single site cohort allows health status?, social systems, education, and health policy to be relatively homogeneous, and therefore the confounding effects of these variables in our study are significantly reduced. Our cohort includes a large number of women and minorities who are not well-represented in other studies. This large participation of women allows for a relatively well-balanced sample to pursue gender analyses and to uncover the enhanced risks of HIV+ hazardous alcohol user females. As the face of the alcohol abuse epidemic is changing with women's rate rising, the need to understand gender differences has never been more necessary; particularly as women are now consuming not only larger amounts, but also different types of alcoholic beverages. The detailed information regarding alcohol intakes provides invaluable data to comprehensively analyze risks by type of alcoholic beverage. Thanks to these advantages, this study allows us to identify populations at high risk of hypertension, including liquor users, women, and smokers that need to be prioritized in prevention campaigns. Interventions to reduce alcohol and smoking among PLWH are of prime importance both for individual prognosis and public health success.
